# Antibody–Biopolymer Conjugates in Oncology: A Review

**DOI:** 10.3390/molecules28062605

**Published:** 2023-03-13

**Authors:** Vivek P. Chavda, Pankti C. Balar, Divya Teli, Majid Davidson, Joanna Bojarska, Vasso Apostolopoulos

**Affiliations:** 1 Department of Pharmaceutics and Pharmaceutical Technology, L M College of Pharmacy, Ahmedabad 380008, Gujarat, India; 2 Institute for Health and Sport, Victoria University, Melbourne, VIC 3030, Australia; 3 Institute of General and Ecological Chemistry, Faculty of Chemistry, Lodz University of Technology, 116 Zeromskiego, 90-924 Lodz, Poland; 4 Immunology Program, Australian Institute for Musculoskeletal Science, Melbourne, VIC 3021, Australia

**Keywords:** antibody biopolymer conjugates, oncology, cancer, antibodies, biopolymers

## Abstract

Cancer is one of the most prevalent diseases and affects a large proportion of the population worldwide. Conventional treatments in the management include chemotherapy, radiotherapy, and surgery. Although being well-accepted, they have many lacunas in the form of severe side effect resulting from lack of targeted delivery. Antibody biopolymer conjugates are a novel method which is an add-on to older methods of immunization. It is used in various diseases and disorders. It ensures the targeted delivery of molecules to increase its efficacy and reduce unwanted effects of the molecule/drug to normal cells. It shows miraculous results in the treatment and management of several cancers even in advanced stages. Herein, we present the chemistry between biopolymer and antibody, their effects on cancer as well as the basic differences between antibody–drug conjugates and antibody–biopolymer conjugates.

## 1. Introduction

Cancer is the leading cause of death worldwide with the latest statistics indicating that 1 in 3 people will develop cancer in their lifetime [1]. To address this issue, the development of innovative therapies is extremely needed. World Cancer Day (celebrated annually on 4 February) is an initiative to unite the entire world together in the fight against this global epidemic. Interestingly, in 1910, Paul Ehrlich proposed the ‘magic bullet’ concept in the context of direct accessing desired targets by effective therapeutic molecules with minimal effects to normal cells [2]. One solution is to identify overexpressed antigens to differentiate healthy cells form cancer cells, with some examples being human epidermal growth factor receptor (HER2) and MUC1 related to the breast cancer or cluster of differentiate 20 (CD20) related to the B cell lymphoma [3,4,5,6]. Monoclonal antibodies (mAbs) were developed to precisely target antigens or receptors to cancer cells and as such, are referred to as targeted therapies. This strategy was advanced following the progress of the hybridoma technology in 1975 [7], and numerous mAbs have been approved. One such example is Herceptin (trastuzumab), a mAb used to primarily treat breast cancer by targeting the HER2 receptor; avastin, rituximab, or cetuximab should not be overlooked. Nevertheless, therapies based on mAbs alone are insufficient, mainly due to low lethality of cancer cells [8]. Therefore, a new concept called antibody biopolymer conjugates (ABC), referring to the product of coupling mAbs with biologic polymers via molecular engineering, has been conceived to develop higher affinity, selectivity, and effectiveness of drugs to be delivered to a target.

**Figure 1:**
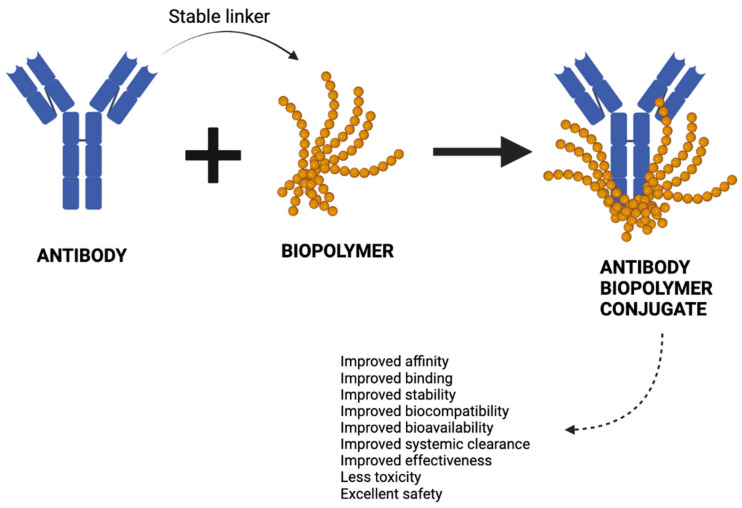
Antibody–biopolymer conjugate (ABC). Monoclonal antibody against a target is humanized and conjugated to a branched high molecular weight biopolymer using a stable linker to develop an ABC.

As such, a recombinant, full length, humanized mAbs is conjugated to a biopolymer. Monomeric units are covalently bonded to larger molecules–polymers. Biopolymers are natural polymers–bio-polymeric molecules derived from cells or extracellular compounds. They predominantly include three types, (i) polynucleotides, (ii) polypeptides, and (iii) polysaccharides and consist of repeating long chains. Biopolymers have many uses in medicine, such as in biomedical engineering, tissue engineering, regenerative medicine, used in medical devices, and are widely used in pharmaceutics and drug delivery. As this approach is a targeted approach, the effectiveness, the half-life, and tissue bioavailability are considerably increased. Improved biocompatibility and fast systemic clearing cannot be neglected. Indeed, a conjugate of a humanized mAb against vascular endothelial growth factor (anti-VEGF) and phosphorylcholine-based biopolymer, results in extended half-life to 10–12 days from 3–4 days of non-biopolymer conjugate [9]. A schematic diagram of ABC is shown in Figure 1.

With their biological effects and proven therapeutic applications of ABC in the field of ophthalmic disease, it is a major step forward for the healthcare system. In the conventional method, the frequency of dosage and the continuous updating of injections along with the economic burden disturbs the life of patients. Therefore, a safer and more effective alternative is required which has a longer duration of action along with patient compliance. Research corresponding to effectiveness of ABCs in the field of oncology has been flourishing tremendously in the last decade. 

Herein, we emphasize the chemistry involved in the current development of ABCs and its advantages over traditional methods of drug delivery systems, with special emphasis in oncology. Their clinical applications in the field of cancer treatment are also highlighted.

## 2. Antibody–Biopolymer Conjugation Chemistry

Antibody–polymer/biopolymer conjugations, directed against tumor-associated antigens, have been an area of much curiosity for some time [10]. ABCs have been developed to improve efficacy, minimize toxicity, and to attain site-specificity and excellent safety [10,11].

**Figure 2:**
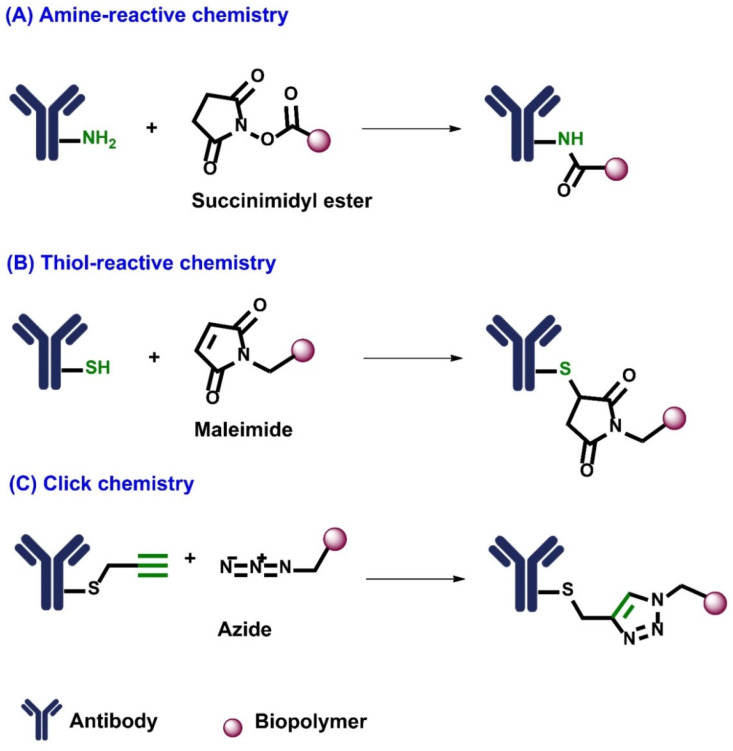
Bioconjugation chemistry: (**A**) amine-reactive chemistry, (**B**) thiol-reactive chemistry, (**C**) click chemistry.

The novel drug delivery system, which is composed of polymers or biopolymers and their conjugates with antibodies or fragments of antibodies are suitable as carriers and as anti-cancer and anti-inflammatory agents [12]. Antibodies are usually coupled to biopolymers using bioconjugation method. Bioconjugation is a chemical reaction between two molecules (at least one of which should be a biomolecule) to form a stable covalent linkage [13]. The advantage of applying conjugates lies in the selective delivery to the target site and in the possible protection of antibodies against fast enzymatic degradation and excretion, thereby leading to a higher antibody concentration against the tumorigenic antigen [14]. Moving forward, bioconjugation methods are highly site-specific and cause minimal perturbation to the active form of the biomolecules [15]. Classical bioconjugation reactions include second order reactions which rely on the reactivity of functional groups present in protein, peptide, or sugar-like biomolecules. Cysteine and lysine residues of proteins are commonly encountered for site-specific bioconjugation which contains thiol and amino functional groups, respectively [16]. Because of its high chemo selectivity, click chemistry has become an efficient strategy for modifying functionality of biomolecules [17]. By virtue of the admirable stability of amide linkages, they become attractive for bioconjugation [18]. A biopolymer containing carboxylic acid group can be treated with coupling agent such as ethyl(dimethylaminopropyl) carbodiimide (EDC) and N-hydroxysuccinimide (NHS) to activate carboxylic acid into corresponding N-hydroxysuccinimidyl ester, which will be treated with amino groups of lysine side chains and N-terminus of peptide to form amide bonds [18] (Figure 2A). Thiols are more potent nucleophiles than amino groups in aqueous solutions. As cysteine is the second least common amino acid in natural proteins, the derivatization of a cysteine residue is a popular method of bioconjugation [19]. The thiol-reactive functional groups include maleimides, bisulfides, and haloacetamides. Amongst them, maleimides are widely used electrophiles for thiol-mediated bioconjugation. Thiol-containing antibodies undergo Michael addition with maleimide-bearing biopolymers to afford succinimidyl thioethers (Figure 2B) [20]. Biologic oligomers and biopolymers, such as carbohydrates, peptides, and nucleic acids, have been modified by using the copper-catalyzed azide-alkyne cycloaddition click reaction (Figure 2C) [17].

Antibody–biopolymer conjugates are also known as biopolymeric prodrugs, which are anticipated as novel drug delivery systems formulated for the incorporation of therapeutic agents into biopolymers of choice using selected functionalities [21]. Antibodies serve a dual purpose here—as therapeutic agent and as targeting moieties to attain the site specificity [22]. These biopolymeric prodrugs fit in with current trends oriented towards natural sources. Biopolymers are natural polymers produced by the cells of living organisms which can be classified according to the monomers used, such as polynucleotides, polypeptides, and polysaccharides. Chitosan, hyaluronic acid, dextran, heparin, silk fibroin, pullulan, or polysaccharides from *Auricularia auricula* are good examples of natural polymers employed in biopolymeric prodrugs [23]. They have widely been exploited for the delivery of therapeutic agents due to their outstanding biocompatible and biodegradable characteristics [24], representing either a valuable or renewable source [23]. Thus, therapeutic agents such as antibodies are chemically conjugated with various biopolymers and the resulting conjugates could slowly elute their active ingredients following cleavage of the biopolymer–antibody linkages under physiological conditions. These conjugates offer several advantages over their therapeutic precursors such as enhancement of water solubility and bioavailability, improvement of pharmacokinetic and biodistribution profiles of antibodies, protecting them from deactivation, and facilitating their transport to the targeted sites [25]. 

It can be mentioned that the selective activity is provided via key characteristics of neoplastic pathology, including inter alia specific enzymes, hypoxia, and extracellular pH. Interestingly, the strategic concept of utilizing specific triggers for the activation of prodrugs, in terms of antibody-directed prodrug therapy, gene-directed enzyme prodrug therapy, and virus-directed enzyme prodrug therapy, was introduced in the late 1980s [24,25]. Next, a new mechanism to activate drugs, depending on the stimuli, known as polymer-directed enzyme drug therapy was developed. Here, the polymer–enzyme conjugates increase the selective release of drug from a polymer conjugate. More specifically, a new polymer enzyme-loaded nanoreactor to release the active drug from prodrug was reported [26,27]. This technology was used in liposomal formulations, known as polymer enzyme liposome therapy, in which the drug molecule is released from the liposome into the tumor site [28].

Table 1 summarizes some examples of antibody–biopolymer conjugates and their efficacy towards several cancers. Poly (β-L-malic acid) and polyethylene glycol (PEG)–antibody conjugates by covalently incorporating anti-HER2/neu peptide (AHNP) (trastuzumab-mimetic 12-merpeptide) for the treatment breast cancer [26]. Jun Xiao et al. synthesized glycol chitosan (GC) and gemcitabine conjugate using NHS/EDC method, which was further conjugated with chitosan antibody and anti-EGFR antibody to afford ABC-GC-Gemcitabine nano bioconjugates. It was reported that these conjugates were able to reduce pancreatic cancer cell proliferation and colony formation, and also inhibited the migration and invasion of SW1990 cells. Refs. [27,28] proposed a microfluidic-assisted approach using a polydimethylsiloxane (PDMS) Y-shaped microreactor for the covalent conjugation of Trastuzumab (TZB), a recombinant antibody targeting HER2 (human epidermal growth factor receptor 2), to doxorubicin-loaded PLGA/Chitosan NPs (PLGA/DOX/Ch NPs) using EDC and N-hydroxysulfosuccinimide (sNHS) mediated bioconjugation reactions. The conjugate showed promising results when checked in vitro against HER2+ breast cancer cells [28]. Rong Zhu et al. carried out a study where CD147 monoclonal antibody was coupled with a complex of α-hederin (α-hed) and chitosan (CS) nanoparticles (NPs) using NHS and EDC. The researchers reported that the half-maximum inhibiting concentration (IC_50_) of α-Hed-CS-CD147-NPs in human liver cancer cell lines HepG2 and SMMC-7721 was lower than that of free α-Hed and α-Hed-CS-NPs [29].

## 3. Current Developmental Status of ABC in Oncology

Monoclonal antibodies are widely used in the treatment and management of cancer patients. However, due to lack of selectivity, it leads to tremendous toxicity. The conjugation with biopolymers enables the selective delivery of antibodies by having a specific affinity towards some over-expressed or lacking receptors on tumor cells [30]. Polymers can form monomolecular structures [31] or micelles [32] to perform their tasks (Table 2). Vladimir P. Torchilin and co-workers stated that 2C5 antibody conjugated with taxol micelles successfully reduced the tumor mass in the heart in both in vivo and in vitro studies [33]. Micellar complex recognizes the surface of the tumor but not the normal cell, hence imparting selectivity of antibody delivery. It also facilitates the delivery of aquaphobic pharmaceuticals [34]. 

In addition, mAb-ch735 was shown to specifically target cancer cell membranes. This targeted approach can allow antibodies to be rapidly internalized into endosomes and lysosomes [35]. Further, the development of nanotechnology has provided an added advantage to ABC. As such, the biodegradable tamoxifen antibody-conjugated polymeric nanoparticles have a targeted delivery of antibodies against breast cancer [36]. α-hederin chitosan conjugated with CD147 monoclonal antibody escalate the action on liver cells malignancy [22]. Due to the lipophilic profile of α-hederin, it holds low bioavailability and poor oral absorption which can be overcome by entrapping it in biopolymer (here, in chitosan). It is also noted that addition of methyl group in CS increases the anti-tumor property [37]. The hepatocellular cell line CC531 responds extremely well to the mAb CC52-liposome conjugates. In mice, it was shown that even 24 h later, 20–30% liposomes were still present in the bloodstream, showing persistent release to maintain the ideal antibody concentration. The control had a 2-fold greater splenic uptake than the conjugated ones, which lengthens the duration of the effect [38].

**Table 2:** Examples of applications of ABC in oncology.

ABC	Cancer Type	Ref.
2C5 antibody conjugated with taxol micelles	Heart tumor	[33,34]
tamoxifen antibody conjugated with polymeric nanoparticles	Breast cancer	[36]
CD147 conjugated with α-hederin chitosan	Liver cancer	[37]
CC52-liposome conjugate	Colon cancer	[38]
doxorubicin antibody–liposomes	Lung carcinoma	[39]
Cetuximab conjugate with docetaxel loaded poly (γ-glutamic acid) nanoparticle	Gastric cancer	[40]
mAb 19–24 conjugated with daunomycin by dextran	fibrosarcoma	[41]
mAb conjugated with poly (butyl cyanoacrylate)	glioblastoma	[42]

The effectiveness of drug when conjugated with ABC is increased many-fold. It also helps with the pharmacokinetic aspects of the molecule. Liposomes associated with doxorubicin antibody showed a significant effect on murine Lewis lung carcinoma (LLC) and human mammary adenocarcinoma BT-20 cell lines. Within 24 h, the mAb 2C5 lysis was 90% of LLC and 80% of BT-20 cells [39]. Stomach carcinogenesis is on account of overexpression of epidermal growth factor receptor (EGFR) in 27–44% of the initial tumor patient. Cetuximab conjugate with docetaxel loaded poly (γ-glutamic acid) nanoparticle targets on EGRF which arrests the cell division and eventually leads to cell death [40]. The capacity to initiate or regulate drug release in polymeric-based nanoparticles gives them certain benefits over other nanoparticles such as liposomes, such as the possibility for a more powerful medicinal payload and improved stability (liposomal phospholipids are susceptible to oxidation) [30]. Further, in vitro in human fibrosarcoma cells, the mAb 19–24 was efficient when combined with daunomycin using dextran as opposed to being unconjugated [41]. In another study drug–prodrug nanoparticle (PDNP) binding to HepG2 cells was much lower compared to mAb-PDNP. Moreover, poly (butyl cyanoacrylate) (PBCA) nanoparticles conjugated with mAb showed that cytotoxicity was improved by 40% as compared to carboplatin alone. In rats with glioblastoma, the survival was longer when compared to free carboplatin treatment and side effects were significantly reduced in the liver, kidney, and brain compared to carboplatin alone [42]. 

### 3.1. Clinical Studies

Clinical trials are of utmost importance to determine the efficacy and toxicity of drug molecules, and other effects on human health outcomes. KSI-301 is the first-in-class of ABC, consisting of anti-VEGF IgG1 monoclonal antibody and phosphorylcholine-based biopolymer, designed to increase either ocular half-life or tissue bioavailability. It has a high binding affinity. Indeed, in preclinical studies, the half-life was extended (~11 days) in terms of ranibizumab (~3 days) and aflibercept (~4 days). Moreover, KSI-301 can be cleared rapidly. It is currently in human clinical trials against retinal vascular diseases [9]. Nevertheless, it should be highlighted that ABC is a novel approach to enhance clinical effects and, despite being ABC an astounding molecule, human clinical trials are still limited in the field of cancer (Table 3). As such, further studies are required in humans. 

#### Alternative Diagnostic Uses

ABCs also possess imaging qualities with one example being in ovarian cancer. In fact, ovarian cancer can be easily identified using polymeric fluorescent nanoparticles (PFNPs) attributed to their distinctive optical characteristics. Excellent photosensitivity makes PFNPs an innovative tool for the detection and treatment of ovarian cancer cells [43]. In in vitro studies, overexpression of prostate stem cell antigen (PSCA) was identified in 90% of samples where the ability of mAbs to identify PSCA was significantly increased by the use of conjugate–dextran antibodies with superparamagnetic iron oxide [44]. As such, the labeled cell produces intense blue color upon binding with anti-PSCA-dextran-superparamagnetic iron oxide [45]. In non-Hodgkin’s lymphoma (NHL), anti-CD20 polyclonal antibody-radioisotopes/fluorophores conjugated with chitosan has been used for its detection [46]. 

## 4. Discussion

Antibody conjugations provide a novel strategy to produce effective diagnostic and therapeutic (or theranostic) as well as imaging systems. The antibody–biopolymer conjugates are rapidly becoming important compounds and no doubt a revolution in oncology that offers enormous potential and new prospects in ‘drugging the undruggable’ targets. 

### Pros and Cons of ABC

The main advantages of ABCs are as follows: extended half-life/treatment durability, increased tissue bioavailability, a high binding affinity, deeper potency, fast systemic clearance, enhanced tissue penetration, improved stability, efficacy, biocompatibility, and safety [47,48]. Conjugations with polymer nano-delivery systems lead to better solubility and immunological profile [49]. They can deliver poorly soluble chemotherapeutic agents to suppress multiple drug resistance phenomena in tumor cells [48]. Furthermore, ABCs can detect cancer at its earliest stage and induce significant cancer cell death [40,50]. Bioconjugates provide synergistic anti-tumor effects and potency to overcome the complications resulting from chemotherapeutics. Many biopolymers have more complex structures, like the human body; thus, they are better with bodily integration. Moreover, biopolymers, as natural polymers can mimic body parts to sustain normal biological functions. Therefore, they are ideal in biomedical engineering [47]. Diverse efforts have been invested into designing suitable ABC anticancer strategies [51] and several ABCs have been developed in several clinical trials. Nanotechnology is a powerful approach in terms of ABC-based diagnosis and therapy of cancers due to its ease in synthesis, tuneability, and bio-functionalization [46,48]. The unique feature of bio-conjugates is the selective delivery of drugs to pathological sites and the improvement of molecular retention in the blood circulation system [52]. ABCs are stimuli-sensitive and thus, more effective compared to other delivery systems [32]. Importantly, the ABC platform stabilizes the structure of antibodies in terms of favorable clinical features (e.g., low nonspecific interactions and antibody recycling) [48]. Overall, ABCs represent a big step towards perfect diagnosis techniques and therapeutic options–effective, affordable, and with less treatment burden. 

Nevertheless, they are still at the proof-of-concept stage. In vitro efficacy is not ideally translated into clinical effects and the production cost of ABCs is currently high. Thus, ABC therapy is yet not available as a treatment option, especially in underdeveloped countries. In this regard, further studies are required to improve its stability, efficacy, and effectiveness in human clinical trials [52].

## 5. Conclusions

According to the WHO in 2022, cancer has a significant impact in a large population and causes close to 10 million fatalities per year worldwide. The traditional approach of treating cancer has its own disadvantages. Immunization is a crucial component of treating several cancers, yet it has pitfalls such a short half-life and poor pharmacokinetics. However, ABC is an innovative, complex medicinal chemical that enhances the sanative effects of antibodies. Utilizing antibodies makes it easier to target specific cells and effectively reduces illness. Clinical studies on several compounds that treat cancer are being monitored. ABC has targeted breast cancer in a clinical study since it is the primary cause of mortality from cancer, and the healthcare system is hoping for a favorable outcome. The remarkable properties of ABCs will need to be established through further clinical trials. ABCs have the potential to alter the mode of how drugs are administered, with outstanding therapeutic results and with fewer negative effects.

## Figures and Tables

**Table 1:** Summary of the efficacy of several antibody–biopolymer conjugates on cancer.

Conjugate	Antibody	Biopolymer	Type of Cancer	Molecular Design	Ref.
Trastuzumab-polymalic acid	Anti-p185HER2/neu peptide (trastuzumab-mimetic 12-merpeptide)	Poly (β-L-malic acid)	Breast cancer	Conjugates were prepared by covalently incorporating anti-HER2/neu peptide	[26]
Abc-GC-Gemcitabine	Gemcitabine, anti-EGFR antibody	Glycol chitosan	Pancreatic cancer	Bioconjugation using NHS and EDC	[27]
Transtuzumab-chitosan	Transtuzumab	Doxorubicin-loaded poly lactic-co-glycolic acid (PLGA)/Chitosan	Breast cancer	Covalent derivatization using sNHS and EDC	[28]
α-Hed-CS-CD147-NPs	CD147	Chitosan	Liver cancer	Amide linkage formed between CD147 and α-Hed-CS-NPs using NHS and EDC	[29]

**Table 3:** The available clinical trials on ABC associated with drug molecules to enhance the cytotoxicity and effectiveness on cancer.

Other Id	Study Design	Condition	Number Enrolled	NCT Number
CC1	Single group Assignment, Primary Purpose: Treatment	Solid Tumors	26	NCT01702129
SAKK 24/14	Single group Assignment, Primary Purpose: Treatment	Breast cancer	48	NCT02833766
2018-01160; me17Kasenda2	Single group Assignment, Primary Purpose: Basic Science	Glioblastoma	9	NCT03603379

## Data Availability

Not applicable.

## Sample Availability

Samples of the compounds are not available from the authors.
